# TIMP3 Overexpression Improves the Sensitivity of Osteosarcoma to Cisplatin by Reducing IL-6 Production

**DOI:** 10.3389/fgene.2018.00135

**Published:** 2018-04-20

**Authors:** Xiu-guo Han, Hui-min Mo, Xu-qiang Liu, Yan Li, Lin Du, Han Qiao, Qi-ming Fan, Jie Zhao, Shu-hong Zhang, Ting-ting Tang

**Affiliations:** ^1^Shanghai Key Laboratory of Orthopedic Implants, Department of Orthopedic Surgery, Shanghai Ninth People’s Hospital, Shanghai Jiao Tong University School of Medicine, Shanghai, China; ^2^Institute of Hematology, Xuzhou Medical University, Xuzhou, China; ^3^Department of Hematology, The Affiliated Hospital of Xuzhou Medical University, Xuzhou, China; ^4^Department of Orthopedic Surgery, The First Affiliated Hospital of Nanchang University, Nanchang, China; ^5^Department of Orthopedic Surgery, The Second Affiliated Hospital Zhejiang University School of Medicine, Hangzhou, China; ^6^Department of Orthopedic Surgery, Shanghai First People’s Hospital, Shanghai Jiao Tong University School of Medicine, Shanghai, China

**Keywords:** osteosarcoma, TIMP3, IL-6, cisplatin sensitivity, caspase family

## Abstract

Osteosarcoma is the most common bone cancer in children and adolescents. Tissue inhibitors of metalloproteinases (TIMPs)-3 inhibit matrix metalloproteinases to limit extracellular matrix degradation. Cisplatin is a widely used chemotherapeutic drug used to cure osteosarcoma. Interleukin (IL)-6 and TIMP3 play important roles in the drug resistance of osteosarcoma; however, their relationship in this process remains unclear. This study aimed to explore the role of TIMP3 in the cisplatin sensitivity of osteosarcoma and its underlying molecular mechanisms *in vitro* and *in vivo*. We compared TIMP3 expression levels between patients with cisplatin-sensitive and -insensitive osteosarcoma. TIMP3 was overexpressed or knocked down in the Saos2-lung cell line, which is a Saos2 subtype isolated from pulmonary metastases that has higher cisplatin chemoresistance than Saos2 cells. IL-6 expression, cell proliferation, sensitivity to cisplatin, migration, and invasion after TIMP3 overexpression or knockdown were determined. The same experiments were performed using MG63 and U2OS cells. Subsequently, luciferase-labeled Saos2-lung cells overexpressing TIMP3 were injected into the tibiae of nude mice treated with cisplatin. The results showed that IL-6 inhibited TIMP3 expression in Saos2 and Saos2-lung cells via signal transducer and activator of transcription 3 (STAT3) activation. STAT3 knockdown reversed the effect of IL-6. The expression of TIMP3 was higher in patients with cisplatin-sensitive osteosarcoma than in those with insensitive osteosarcoma. IL-6 expression was downregulated upon TIMP3 overexpression, and upregulated by TIMP3 knockdown. TIMP3 overexpression suppressed cell proliferation and enhanced cisplatin sensitivity by activating apoptosis-related signal pathways and inhibiting IL-6 expression *in vitro* and *in vivo*. In conclusion, cisplatin sensitivity correlated positively with TIMP3 expression, which is regulated by the IL-6/TIMP3/caspase pathway. The TIMP3 pathway could represent a target for new therapies to treat osteosarcoma.

## Introduction

Osteosarcoma is the most common, malignant primary bone tumor in children and adolescents (Kansara et al., 2014; Valery et al., 2015; Gianferante et al., 2017). Over the last 20 years, cisplatin has been commonly used as an anticancer drug to treat osteosarcoma (Jaffe, 2014; Isakoff et al., 2015; Sun et al., 2017). However, there has been no improvement in the survival of patients with drug-resistant osteosarcoma (Lin et al., 2016). To date, the mechanisms of chemoresistance of osteosarcoma have been extensively investigated. A study showed that hypoxia promoted drug resistance in osteosarcoma cells by activating the AMPK signaling pathway (Zhao et al., 2016). Another report revealed that microRNA MiR-34a-5p promoted multi-chemoresistance of osteosarcoma by downregulating the expression of the Delta-like (*DLL*) 1 gene (Pu et al., 2017). Thus, it is necessary to understand the drug-resistance mechanism of osteosarcoma, and to provide novel therapeutic strategies for this disease.

Tissue inhibitors of metalloproteinases are important regulators of MMPs. *TIMP3* is located at 22q12.3 in the human genome. TIMP3 is an insoluble glycoprotein that is produced by most cell types and is sequestered at the cell surface, where it is bound by components of the extracellular matrix (Fata et al., 2001). *TIMP3* is related to genes regulating apoptosis, which have been proposed as tumor suppressors (Xu et al., 2013; Saraiva-Esperon et al., 2014). TIMP3 acts as a tumor suppressor to inhibit tumor growth, invasion, and angiogenesis (Baker et al., 1999; Chetty et al., 2008). Our previous study showed that TIMP3 could regulate osteosarcoma cell migration, invasion, and chemotherapeutic resistance *in vitro* (Han et al., 2016). In addition, TIMP3 expression has been linked to favorable outcomes in head and neck cancer (De Schutter et al., 2009). Thus, the prognostic value of TIMP3 in human cancers should be clarified.

Another study reported elevated levels of tumor necrosis factor α (TNF α) in *Timp3*-knockout mice, which activated nuclear factor kappa B (NF-κB) and IL-6 production, subsequently leading to severe inflammation of the liver (Mohammed et al., 2004). A recent study revealed that the loss of *TIMP3* expression promotes tumor invasion via elevated IL-6 production, and predicts relapse and poor survival in patients with human papillomavirus (HPV)-infected non-small-cell lung cancer (Wu et al., 2012), Our previous studies demonstrated that activation of signal transducer and activator of transcription 3 (STAT3) by IL-6 in mesenchymal stem cells promoted the proliferation, drug resistance, and metastasis of osteosarcoma (Tu et al., 2012, 2016). Therefore, IL-6 and TIMP3 play important roles in the drug resistance of osteosarcoma. However, the relationship between TIMP3 and IL-6 in this process remains unclear.

In this study, we used a highly metastatic osteosarcoma cell line, known as Saos2-lung, a luciferase-labeled Saos-2 subtype derived from pulmonary metastases of nude mice with osteosarcoma (Du et al., 2012). Compared with their parental cells, Saos2-lung cells exhibit increased cell adhesion, migration, and invasion ability, and a high level of chemoresistance (Du et al., 2014; Han et al., 2015). We aimed to investigate the crosstalk between IL-6 and TIMP3, and to determine whether TIMP3 influences cisplatin sensitivity, proliferation, migration, and invasion of osteosarcoma cells to reveal the mechanisms underlying osteosarcoma drug resistance.

## Materials and Methods

### Main Materials

Antibodies against TIMP3 (5673), phospho (p)-STAT3 (9145S), STAT3 (12640S), p-Akt (Akt kinase) (4060S), t (total)-Akt (4685S), B-Cell CLL/lymphoma 2 (Bcl-2) (4223S), Bcl-2 associated X protein (Bax) (5023S), cleaved Caspase-3 (1050S), Caspase-3 (14220S), cleaved caspase-9 (20750S), caspase-9 (9502S), glyceraldehyde-3-phosphate dehydrogenase (GAPDH) (2118S), and secondary antibodies (5151S) were purchased from Cell Signaling Technology, Inc. (CST, Danvers, MA, USA). Cisplatin was purchased from Sigma-Aldrich, Inc. (Darmstadt, Germany, P4394). IL-6 was bought from PeproTech., Inc. (Rocky Hill, NJ, United States, 200-06).

### Cell Culture

Human osteosarcoma Saos2, MG63, and U2OS cells were purchased from the Chinese Academy of Sciences (Shanghai, China). The cell lines were characterized by Genetic Testing Biotechnology Corporation (Suzhou, China) using short tandem repeat (STR) markers. The Saos2-lung cell line, which is a subtype of Saos2 labeled with luciferase and reported by our previous studies (Du et al., 2014; Han et al., 2015), is a highly metastatic and chemoresistant human osteosarcoma cell line derived from pulmonary metastases of nude mice with osteosarcoma. All cells were grown in Dulbecco’s modified Eagle’s medium (DMEM) (Hyclone, Tauranga, New Zealand), supplemented with 10% fetal bovine serum (FBS) (Hyclone) and antibiotics (100 U/mL penicillin, 100 μg/mL streptomycin) in a 5% CO_2_ humidified atmosphere at 37°C. All cell lines were used within 20 passages.

### Patients and Specimens

Patients with osteosarcoma involved in this study were hospitalized in the Department of Orthopedic Surgery, The First Affiliated Hospital of Nanchang University, Jiangxi Province, China between March 2015 and December 2016. Informed consent was obtained from the participants in accordance with the regulatory requirements of our institution, and we conducted the study according to guidelines of the ethics committee of the First Affiliated Hospital of Nanchang University (Ethical Approval Number: NCUMC-20150067). In all cases, the diagnosis of conventional osteosarcoma was established using clinical characteristics, radiological findings, and pathological examination. Participants were randomly selected from a sample library, and included eight patients (six male, two female) with a median age of 38.25 years (range: 20–77 years), among which two patients experienced tumor recurrence after surgery within 1 year. According to the inhibition rate of cisplatin in the tumor susceptibility test, the histological specimens of these patients were divided into two groups: A cisplatin-sensitive group (cisplatin sensitivity rate: 50–90%) and a cisplatin-resistant group (cisplatin sensitivity: 10–30%). The two groups had no significant difference in composition regarding age or sex (*P* > 0.05) (**Table 1**).

**Table 1:** The inhibition rate of cisplatin on osteosarcoma.

Patients	Case No.	Specimen No.	Sex	Age (Year)	Cisplatin sensitivity %
Sensitive patients	001	652875	Male	20	56.83


	002	662588	Male	31	63.12
	003	666959	Male	31	66.21
	004	693281	Female	77	85.92
Resistance patients	005	659856	Male	34	12.32


	006	696185	Female	45	22.83
	007	703686	Male	37	15.62
	008	718065	Male	31	10.21

The inhibition rate of cisplatin in osteosarcoma was detected. Based on these data, the samples were divided into two groups: A cisplatin sensitive group (Case Nos. 001–004) and a cisplatin resistance group (Case Nos. 005–008).

### Enzyme-Linked Immunosorbent Assay

The human IL-6 enzyme-linked immunosorbent assay (ELISA) kit was purchased from R&D systems (Minneapolis, MN, United States, HS600B). Saos2 and Saos2-lung cells (1 × 10^4^ per well) were plated in 6-well plates. On the following day, the medium was replaced with 2 mL per well of fresh serum-free DMEM, and the culture supernatants were collected after 24 h. The IL-6 concentration in the culture supernatants was determined using ELISA according to the manufacturer’s protocols.

### Cell Transfection

For TIMP3 overexpression, Saos2-lung, MG63, and U2OS cells were transfected with green fluorescent protein (GFP)-labeled-lentiviral vectors (GeneChem, Shanghai, China) expressing TIMP3 or a vector with a scrambled control sequence. Cells were subsequently exposed to 3 μg/mL of puromycin (Sigma, Darmstadt, Germany, SBR00017) for 4 weeks post infection. Several clones were generated, and these were expanded and maintained in 1 μg/mL of puromycin to eliminate tumor cells that had lost TIMP3 expression. For TIMP3 knockdown, cells were transfected with a short interfering RNA (siRNA) against *TIMP3*, which could be maintained for 7 days after transfection. The sequences of the siRNA against *TIMP3* were 5′-GCAUAAUC UGAGCCCUGCU-3′ (forward) and 5′-AGCAGGGCUCAGAUUAUGC-3′ (reverse).

Meanwhile, Saos2-lung cells were transfected using Lipofectamine RNAiMAX Reagent (Invitrogen) with a siRNA against *STAT3* (Thermo Scientific, Waltham, MA, United States, siRNA ID: 116558). The sequences of the siRNA against *STAT3* were 5′-GCAGCAGCTGAACAACATGTTCAAGAGACATGTTGTTCAGCTGCTGCTTTTT-3′ (forward) and 5′-AATTAAAAAGCAGCAGCTGAACAACATGTCTCTTGA ACATGTTGTTCAGCTGCTGCTGCGGCC-3′ (reverse). After transfection for 72 h, the cells treated with or without IL-6 were harvested for protein assays.

### RNA Isolation and Real-Time PCR

Total RNA was isolated from Saos2-lung, MG63, U2OS cells (overexpressing TIMP3 or with TIMP3 knockdown), and related controls using an RNeasy Mini Kit (Qiagen, Dusseldorf, Germany), and cDNA was synthesized using an iScript cDNA Synthesis Kit (Bio-Rad, Hercules, CA, United States). Subsequently, *IL6* and *TIMP3* expression levels were assessed using an ABI 7500 Sequence Detection System (Thermo Scientific, Waltham, MA, United States) and SYBR Premix Ex Taq (Takara, Dalian, Liaoning, China). All procedures were performed according to the manufacturer’s protocols. The mRNA levels were normalized to those of the housekeeping gene *GAPDH*. The primer sequences are listed in **Table 2**.

**Table 2:** Sequences of primers used in real-time PCR.

Gene		Primer sequences (5′-3′)
IL-6	Forward	TCAATATTAGAGTCTCAACCCCCA
	Reverse	GAGAAGGCAACTGGACCGAA
TIMP3	Forward	CGATGAGGTAATGCGGCTCT
	Reverse	CATCTTGGTGAAGCCTCGGT
GAPDH	Forward	GAAATGAATGGGCAGCCGTT
	Reverse	GAGTTAAAAGCAGCCCTGGTG

### Western Blotting

Total protein was isolated from osteosarcoma cells with overexpression or knockdown of TIMP3 and their related controls, before or after cisplatin treatment. Whole cell extracts were prepared by lysing the cells in radioimmunoprecipitation assay (RIPA) buffer (150 mM NaCl, 1% sodium deoxycholate, 0.1% SDS, 50 mM Tris-HCl pH 7.4, 1 mM ethylenediaminetetraacetic acid (EDTA), 1 mM phenylmethylsulfonyl fluoride (PMSF), and 1% Triton X-100) containing a cocktail of protease and phosphatase inhibitors. Equal amounts of protein (30 μg) were separated by sodium dodecyl phosphate-polyacrylamide gel electrophoresis (SDS-PAGE), and transferred to polyvinylidene fluoride (PVDF) membranes. The membranes were probed with primary antibodies against TIMP3, p-STAT3, STAT3, p-Akt, t-Akt, BCL-2, BAX, Cleaved Caspase-3, Caspase-3, Cleaved Caspase-9, Caspase-9, and GAPDH. The target proteins were detected using the Odyssey Infrared Imaging System (LI-COR Biosciences, Lincoln, NE, United States).

### Analysis of Cell Proliferation and Cisplatin Sensitivity

A Cell Counting Kit-8 (CCK-8; Dojindo, Tokyo, Japan) was used to quantitatively evaluate cell viability. Cell proliferation assays were performed after the overexpression or knockdown of TIMP3 in Saos2-lung, MG63, and U2OS cells. Approximately, 1000 cells were seeded into each well of a 96-well plate. Each day for 7 days, culture medium was removed and the cells were washed with phosphate-buffered saline (PBS) before the addition of 100 μl DMEM and 10 μl CCK-8 solution to each well and incubation at 37°C for 2.5 h. The optical density at 450 nm was determined daily using a microplate reader (BioTek, Winooski, VT, United States). Values obtained for the treatment groups were divided by those of their corresponding controls to obtain the ratio of viable cells.

Cisplatin sensitivity analysis was performed after the overexpression or knockdown of TIMP3 in Saos2-lung cells. Approximately 5000 cells were seeded into each well of a 96-well plate, and treated with 0, 1, 3, 5, or 10 μg/mL cisplatin for 96 h after adherence. The cisplatin concentrations used were based on clinical treatment programs (20 mg/m^2^ ≈ 5 μg/mL). Cell proliferation assays using CCK-8 kits were performed as described above at 24, 48, 72, and 96 h.

### Analysis of Apoptosis

Apoptosis of Saos2-lung with overexpression or knockdown of TIMP3 and their related controls was measured by flow cytometry using Annexin V/Propidium Iodide (PI) double-immunofluorescent staining. Cells were seeded at 2 × 10^5^ cells per well in 6-well plates, and harvested 24 h after treatment with 5 μg/mL cisplatin. The cells were resuspended in 1 × annexin-binding buffer to verify the rates of drug-induced apoptosis using an Annexin V-fluorescein isothiocyanate (FITC)/PI Kit (Bender Medsystems, Burlingame, CA, United States) according to the manufacturer’s protocols. Apoptotic events were analyzed according to a previous study (Han et al., 2015).

The cell nuclei of dead cells were detected by Hoechst staining in Saos2-lung cells with overexpression or knockdown of TIMP3 and their related controls after treatment with cisplatin.

### Animal Models

Four-week-old nude mice (BALB/c, nu/nu; SIPPR-BK Laboratory Animal Co., Ltd., Shanghai, China) were housed under pathogen-free conditions at 26–28°C and 50–65% humidity. All animal operations were approved by the Animal Ethics Committee of the Shanghai Jiao Tong University School of Medicine (Ethical approval number: A-2016-017). For intratibial injections, luciferase-labeled Saos2-lung cells transduced with TIMP3 lentiviral particles or particles containing a scrambled control sequence were harvested, counted, and resuspended in PBS to a final concentration of 2 × 10^8^ cells/mL. Trypan blue exclusion testing showed that the cells were >95% viable before injection. The animals were anesthetized with 3.5% pentobarbital, and 1 × 10^7^ cells resuspended in 50 μl of PBS were injected into the proximal tibia using a 25-gauge needle. After the tumors had reached 100 mm^3^ in size, the mice of the experimental group were injected intraperitoneally twice per week with cisplatin (15 mg/kg), whereas the mice of the control group were treated with normal saline. The tumor volumes were calculated using the formula: volume = 0.2618 × L × W × (L + W), where W and L represent the average width and length of the tumor, respectively (Han et al., 2015).

### Bioluminescence Assay

For *in vivo* imaging, mice were injected intraperitoneally with 200 μl of 15 mg/mL luciferin (Keyuandi, Shanghai, China) 7–8 min before anesthetization with isoflurane. The images were obtained and analyzed according to a previous study (Han et al., 2015).

### Immunohistochemistry and TUNEL Assay

Sections with a thickness of 5 μm were cut from the specimens taken from the patients and embedded in paraffin after formalin fixation, and stained immunohistochemically for TIMP3. The immunohistochemical staining protocol was the same as that used in a previous study (Han et al., 2015).

In the animal experiment, the mice were sacrificed after 5 weeks of cisplatin treatment. Tumor samples from each nude mouse were fixed in 4% paraformaldehyde and subjected to immunohistochemical staining using the same procedure as that used for the patient samples. Immunohistochemical staining of TIMP3, cleaved Caspase-3, p-Akt, proliferating cell nuclear antigen (PCNA) (also known as CST), and IL-6 was carried out according to a previous study (Han et al., 2015). Apoptosis was assessed using a terminal deoxynucleotidyl transferase dUTP nick-end labeling kit (TUNEL; Roche Applied Science, Mannheim, Baden, Germany) according to the manufacturer’s instructions. Nuclei were counterstained with 4′,6-diamidino-2-phenylindole (DAPI).

### Migration and Invasion Assays

The migration and invasion abilities of Saos2-lung cells with TIMP3 overexpression or knockdown, and their related controls, were measured using Transwell assays. Approximately, 1 × 10^5^ cells were seeded onto a Matrigel-coated polycarbonate membrane insert (6.5-mm diameter, 8.0-μm pores) in a Transwell apparatus (Costar, Cambridge, MA, United States), and maintained in DMEM containing 0.2% bovine serum albumin (BSA). DMEM containing 10% FBS was added to the lower chamber. The migration and invasion assays were then performed and analyzed according to a previous study (Han et al., 2016).

### Statistical Analysis

Statistical analysis was performed using the SPSS statistical software program Version 15.0 (SPSS Inc., Chicago, IL, United States). Data are represented as means ± standard deviation (SD). Comparisons between two groups were performed using Student’s *t*-test, and one-way analysis of variance (ANOVA) was used for multiple comparisons. Each sample was analyzed in triplicate, and the experiments were repeated three times. *P* < 0.05 was considered to indicate a statistically significant difference.

## Results

### IL-6 Inhibited TIMP3 Expression via STAT3 Activation

**FIGURE 1:**
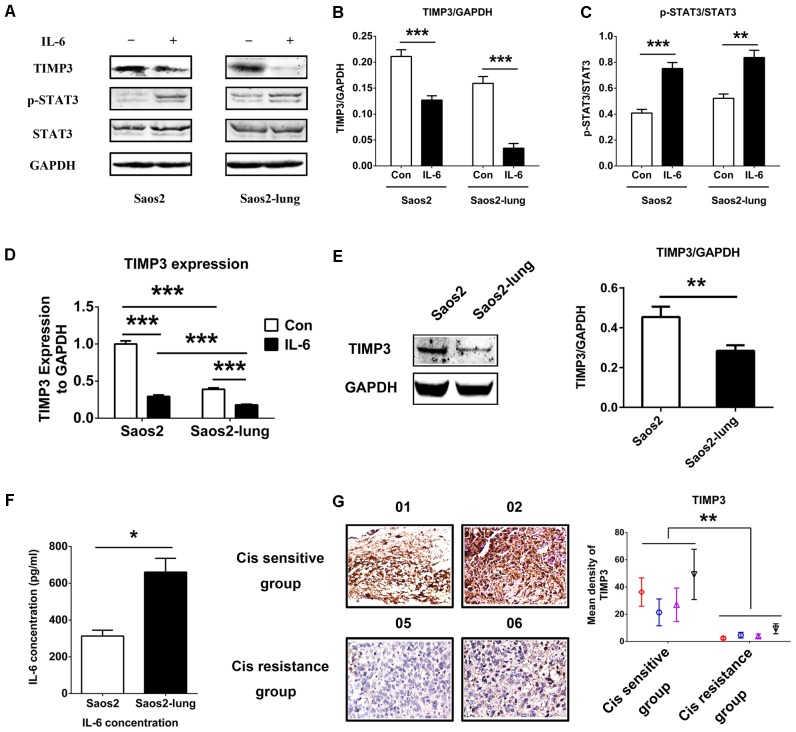
IL-6 inhibits TIMP3 expression through STAT3 activation and TIMP3 is upregulated in the cisplatin sensitive group. **(A)** Western blotting analysis of TIMP3, p-STAT3, and STAT3 protein levels in extracts from Saos2 and Saos2-lung cells treated with 20 ng/mL IL-6 for 24 h. **(B,C)** The results are expressed as the ratio of TIMP3 to GAPDH, and p-STAT3 to STAT3. **(D)** The expression of *TIMP3* was measured in Saos2 and Saos2-lung cells with or without IL-6 treatment via real-time PCR, with *GAPDH* as a housekeeping gene. **(E)** The expression of TIMP3 was measured in Saos2 and Saos2-lung cells via western blotting, with GAPDH as an internal reference. **(F)** Sub-confluent Saos2 and Saos2-lung cells were grown in serum-free DMEM for 24 h, and the IL-6 level in the supernatant was detected using an ELISA. **(G)** Immunohistochemical analysis of TIMP3 was carried out in the tumor samples from different patients. Data are presented as the means ± SD. ^∗∗^*P* < 0.01, ^∗^*P* < 0.05. All data were obtained from at least three independent experiments.

To study the relationship between IL-6 and TIMP3, we used western blotting to detect the levels of TIMP3 and p-STAT3 in Saos2 and Saos2-lung cells treated with 20 ng/mL of IL-6 for 24 h. Upon stimulation by IL-6, the level of TIMP3 was downregulated, accompanied by the activation of p-STAT3 (**Figures 1A–C**). PCR analysis also showed that IL-6 could inhibit *TIMP3* expression (**Figure 1D**). In addition, *TIMP3* expression was higher in Saos2 cells than in Saos2-lung cells (**Figure 1D**), and this was confirmed by western blotting analysis (**Figure 1E**). Furthermore, the autocrine effects of IL-6 were tested using an ELISA, and the results showed that Saos2-lung cells secreted more IL-6 than Saos2 cells (**Figure 1F**). Taken together, these data suggested that IL-6 could inhibit TIMP3 expression in Saos2 and Saos2-lung cells through activation of STAT3.

Osteosarcoma cells from patients exhibited different levels of sensitivity to cisplatin treatment. According to the inhibitory effects of cisplatin on tumor cells, all osteosarcoma samples were divided into two groups: A cisplatin-sensitive group (samples 001–004) and a cisplatin-insensitive group (samples 005–008) (**Table 1**). The expression profile of TIMP3 was then analyzed by immunohistochemical staining in the two groups. As shown in **Figure 1G**, intense immunoreactivity of TIMP3 was observed in cisplatin-sensitive samples (patients 001–004). On the contrary, low expression of TIMP3 was observed in samples from cisplatin-insensitive patients (patients 005–008). Interestingly, the expression of TIMP3 in the highly chemoresistant Saos2-lung osteosarcoma cells was also lower than that in the parental Saos2 cells, as assessed by PCR and western blotting analysis (**Figures 1D,E**). Therefore, these data suggested that cisplatin sensitivity correlated positively with TIMP3 expression.

### IL-6 Expression and STAT3 Phosphorylation Correlated Negatively With TIMP3 Expression

**FIGURE 2:**
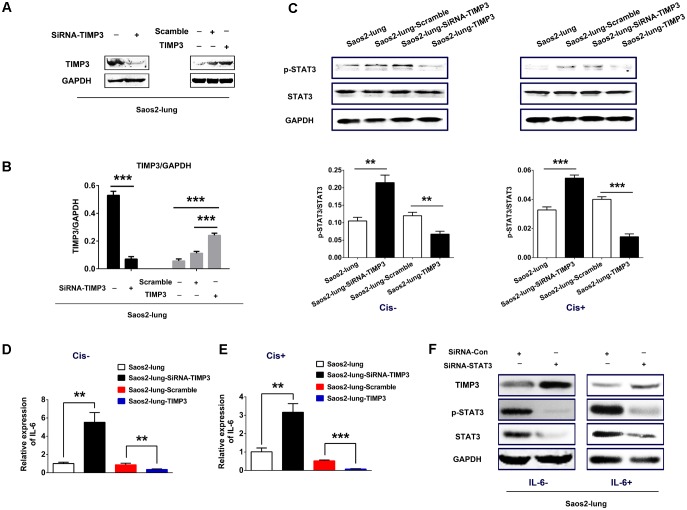
IL-6 and p-STAT3 expression after the overexpression or knockdown of TIMP3 in Saos2-lung cells. **(A)** The identification of TIMP3 overexpression and knockdown in Saos2-lung cells. **(B)** The results are expressed as the ratio of the expression of the target gene to that of *GAPDH*. **(C)** The expression levels of p-STAT3 and STAT3 were measured in transfected-Saos2-lung cells treated with or without cisplatin via western blotting, with GAPDH was an internal reference. The results are expressed as the ratio of p-STAT3 to STAT3. **(D)** *IL6* gene expression in transfected-Saos2-lung cells without cisplatin treatment. **(E)** *IL6* gene expression in transfected-Saos2-lung cells with cisplatin treatment. **(F)** The expression level of TIMP3, p-STAT3, and STAT3 was measured in STAT3 knockdown Saos2-lung cells with or without IL-6. Data are presented as the means ± SD. ^∗∗∗^*P* < 0.001, ^∗∗^*P* < 0.01. All data were obtained from at least three independent experiments.

Saos2-lung, MG63, and U2OS cells were transduced with either lentiviral particles containing the *TIMP3* cDNA or a vector with a scrambled control sequence, or a siRNA directed against TIMP3. Western blotting demonstrated the successful TIMP3 overexpression or knockdown in Saos2-lung (**Figures 2A,B**), MG63, and U2OS (Supplementary Figure 2A) cells.

To confirm the connection between IL-6 and TIMP3, we detected *IL6* expression in transfected Saos2-lung cells with or without cisplatin treatment using real-time PCR. The results showed that the expression of *IL6* was negatively correlated with TIMP3 expression, regardless of cisplatin treatment (**Figures 2D,E**). Similar results were obtained in transfected MG63 and U2OS cells (Supplementary Figure 2B). We then analyzed the phosphorylation of STAT3, which is a downstream signal protein of IL-6. The results showed that phosphorylation of STAT3 was negatively correlated with TIMP3 expression, regardless of cisplatin treatment (**Figure 2C**). Furthermore, we transfected a siRNA against *STAT3* into Saos2-lung cells to confirm the link between IL-6/p-STAT3 and TIMP3 downregulation. We found that the inhibitory effect of IL-6 on TIMP3 was blocked by STAT3 knockdown (**Figure 2F**). In conclusion, the expression of IL-6 and the abundance of p-STAT3 were negatively correlated with TIMP3 expression.

### TIMP3 Expression Was Negatively Associated With Osteosarcoma Progression

**FIGURE 3:**
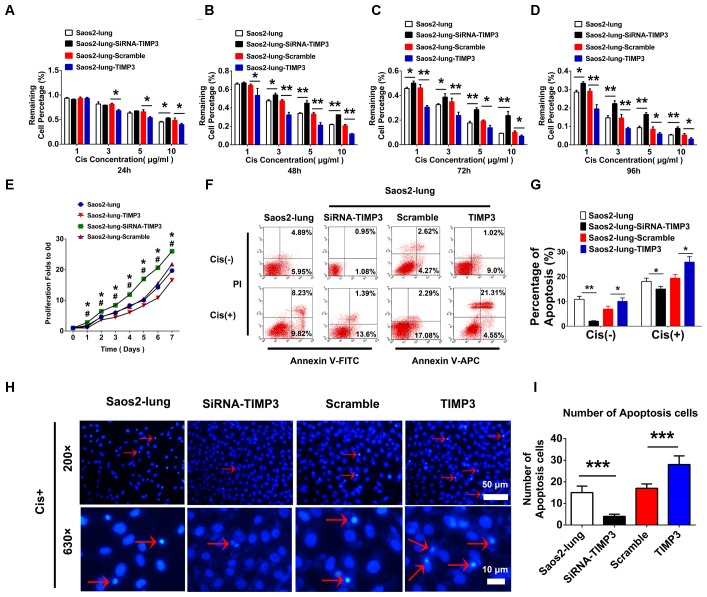
Cell proliferation and cisplatin sensitivity after the overexpression or knockdown of TIMP3 in Saos2-lung cells. **(A–D)** Cisplatin sensitivity of transfected-Saos2-lung cells at 24, 48, 72, and 96 h. **(E)** Cell proliferation in transfected-Saos2-lung cells without cisplatin. **(F)** Flow cytometry assessment of apoptosis in transfected-Saos2-lung cells without or with cisplatin treatment. **(G)** Apoptosis rates of transfected-Saos2-lung cells with or without cisplatin treatment. **(H)** Hoechst staining of transfected-Saos2-lung after cisplatin treatment for 24 h. **(I)** The quantification of hoechst staining. The results are expressed as the ratio of p-Akt to t-Akt. Data are presented as the means ± SD. ^∗^*P* < 0.05 between the Saos2-lung and siRNA groups, #*P* < 0.05 between the Saos2-scramble and Saos2-lung-TIMP3 groups, separately, in a cell proliferation assay. ^∗∗∗^*P* < 0.001, ^∗∗^*P* < 0.01, ^∗^*P* < 0.05. All data were obtained from at least three independent experiments.

The proliferation of Saos2-lung, MG63, and U2OS cells with TIMP3 overexpression was inhibited from 1 to 7 days after transfection. Conversely, knockdown of TIMP3 promoted the proliferation of Saos2-lung cells from the 1st day post transduction (**Figure 3E** and Supplementary Figures 2C,D). These findings indicated that TIMP3 expression was negatively correlated with osteosarcoma cell proliferation *in vitro*. The Transwell assay also showed that TIMP3 expression was negatively correlated with osteosarcoma cell migration and invasion (Supplementary Figures 1, 3). We then examined the role of TIMP3 in the resistance of osteosarcoma cells to cisplatin using CCK-8 assays and Saos2-lung cells overexpressing or knocked down for TIMP3. TIMP3 overexpression at different levels inhibited the viability of Saos2-lung at 24, 48, 72, and 96 h after treatment with 1, 3, 5, and 10 μg/m cisplatin (**Figures 3A–D**). Moreover, TIMP3 knockdown increased the viability of Saos2-lung cells after the same treatment (**Figures 3A–D**). We then examined the role of TIMP3 in cisplatin-induced apoptosis of Saos2-lung cells post transfection. Compared with the scrambled control, TIMP3 overexpression enhanced the rate of cisplatin-induced apoptosis in Saos2-lung cells (19.37% ± 1.52% *vs*. 25.86% ± 2.21%) (*P* < 0.05) (**Figures 3F,G**). Compared with the control, TIMP3 knockdown significantly decreased cisplatin-induced apoptosis in Saos2-lung cells (18.05% ± 1.34% *vs*. 14.99% ± 1.06%) (*P* < 0.05) (**Figures 3F,G**). Nuclei staining also showed that a large number of apoptotic cells were present among the cells overexpressing TIMP3 after cisplatin treatment (**Figures 3H,I**). These results indicated that TIMP3 expression was positively associated with the sensitivity of osteosarcoma cells to cisplatin *in vitro*. Taken together, TIMP3 expression was negatively associated with osteosarcoma progression.

### TIMP3 Overexpression Enhanced the Activation of Apoptosis-Related Signaling in Osteosarcoma Cells

**FIGURE 4:**
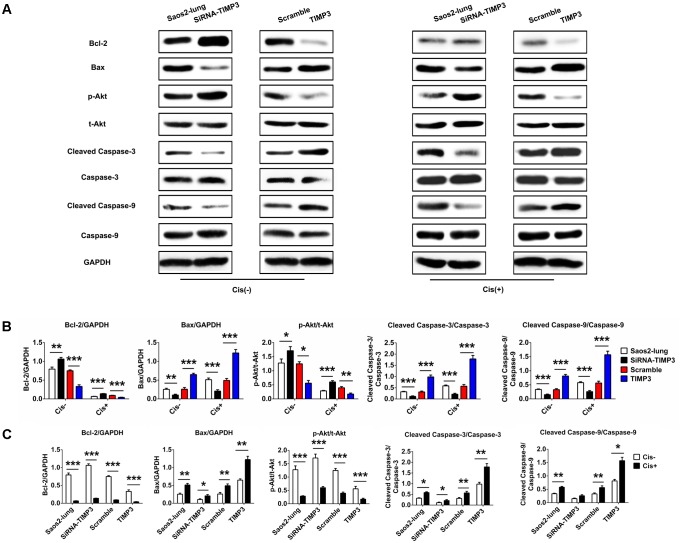
Apoptosis-related gene expression in Saos2-lung cells following overexpression or knockdown of TIMP3. **(A)** The expression levels of Bcl-2, Bax, p-Akt, cleaved caspase-3, and cleaved caspase-9 were measured using western blotting in transfected-Saos2-lung cells, with or without cisplatin. **(B,C)** The results are expressed as the ratio of Bcl-2 to GAPDH, Bax to GAPDH, p-Akt to t-AKT, cleaved caspase-3 to caspase-3, and cleaved caspase-9 to caspase-9. Data are presented as the means ± SD. ^∗∗∗^*P* < 0.001, ^∗∗^*P* < 0.01, ^∗^*P* < 0.05. All data were obtained from at least three independent experiments.

To identify the underlying molecular mechanisms, we used western blotting to analyze the levels of Bcl-2, Bax, p-Akt, cleaved caspase-3, and cleaved caspase-9, which are very important in the regulation of apoptosis in osteosarcoma cells following cisplatin treatment. TIMP3 overexpression in Saos2-lung cells inhibited the activation of Akt and Bcl-2, and increased the levels of Bax, cleaved caspase-3, and cleaved caspase-9 following cisplatin treatment. TIMP3 knockdown had antagonistic effects (**Figure 4**). Taken together, we concluded that TIMP3 overexpression in osteosarcoma cells may directly or indirectly increase the expression and/or activity of the caspase family proteins, eventually resulting in improved sensitivity to cisplatin.

### TIMP3 Overexpression Enhanced the Chemotherapy Sensitivity of Saos2-Lung Cells to Cisplatin *in Vivo*

**FIGURE 5:**
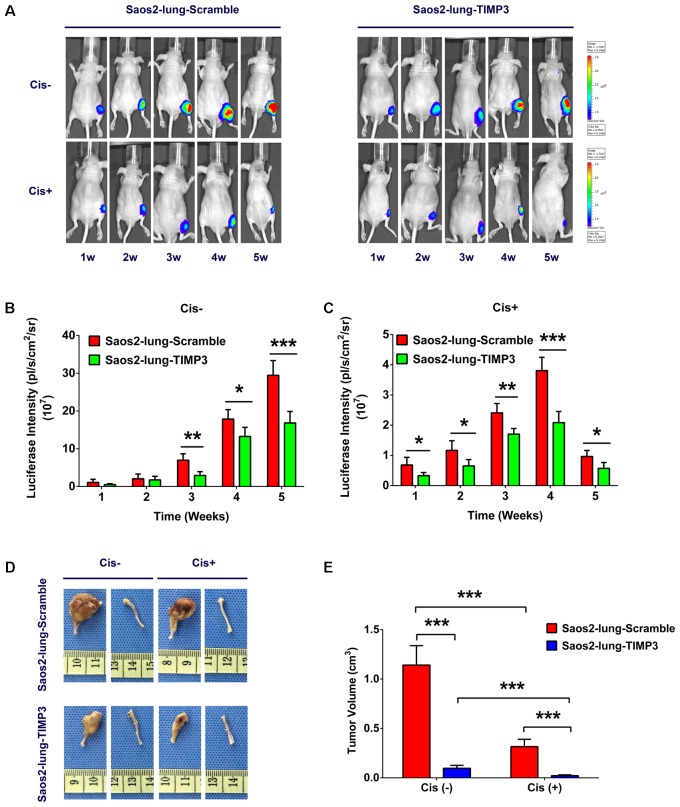
Cisplatin sensitivity of Saos2-lung cells after TIMP3 overexpression *in vivo*. **(A–C)** Cisplatin or saline was used to treat nude mice bearing osteosarcoma cells. Saos2-lung cells with and without TIMP3 overexpression were labeled with luciferase and implanted in the tibia. The tumor volume was evaluated via *in vivo* bioluminescence assay at 1–5 weeks after cisplatin or saline treatment. **(D,E)** The mice were sacrificed, and the tumor volume was measured and calculated during the 5th week after treatment. The data are presented as the means ± SD. ^∗∗∗^*P* < 0.001, ^∗∗^*P* < 0.01, ^∗^*P* < 0.05. Each group contained five animals.

TIMP3 was constitutively overexpressed because of lentivirus transfection, and siRNA did not produce this effect; therefore a nude-mouse model of primary osteosarcoma was established by injecting Saos2-lung cells with and without TIMP3 overexpression into the bone marrow cavity of mouse tibiae. Treatment with cisplatin was considered experimental, and treatment with saline was considered to be the control. To study the effect of cisplatin on the growth of Saos2-lung tumor cells with and without TIMP3 overexpression in a dynamic manner, we labeled Saos2-lung cells with luciferase in advance. At 1, 2, 3, 4, and 5 weeks after cisplatin treatment, the tumor volume was evaluated via an *in vivo* bioluminescence assay. The results showed that compared with saline treatment, cisplatin treatment led to a progressive decrease in luciferase intensity. In contrast, the luciferase intensity in Saos2-lung cells with TIMP3 overexpression decreased substantially compared with that in Saos2-lung cells without TIMP3 overexpression, as detected by the *in vivo* bioluminescence assay (**Figures 5A–C**). At 5 weeks after cisplatin treatment, the mice were sacrificed, and the tumor volume was measured. Cisplatin had decreased the tumor volume. In comparison, the volume of the tumor formed by Saos2-lung cells with TIMP3 overexpression had decreased considerably compared to that of the scramble group (**Figures 5D,E**), which was in accordance with our *in vitro* data. Therefore, TIMP3 overexpression could enhance the sensitivity of Saos2-lung cells to cisplatin *in vivo*.

### Immunohistochemistry of Tumor Tissues

**FIGURE 6:**
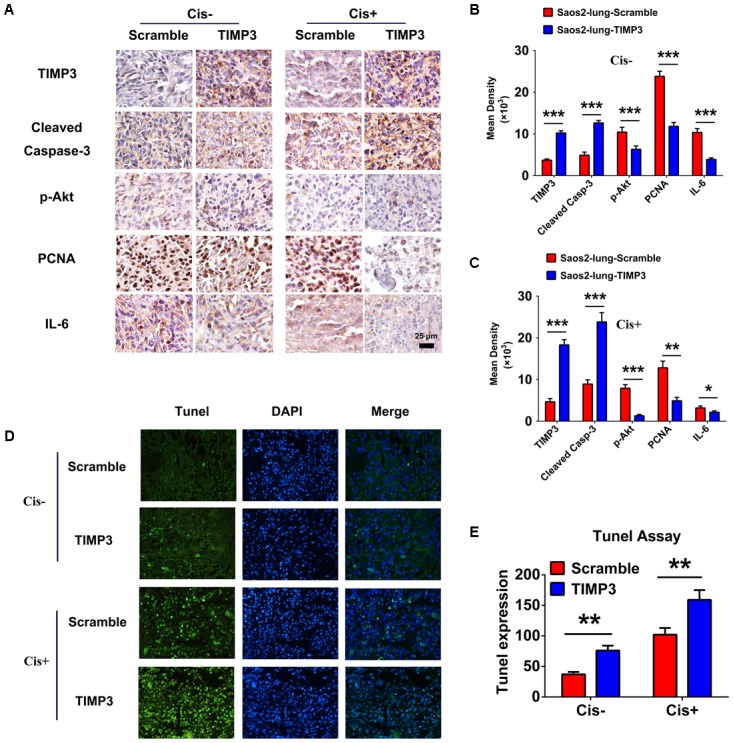
Immunohistochemical analysis of tumor samples with and without TIMP3 overexpression. **(A)** The expression profiles of TIMP3, cleaved caspase-3, p-Akt, PCNA, and IL-6 were evaluated using immunohistochemistry. **(B,C)** Quantification of positive staining was performed using Image-Pro Plus 6.0 software. The results are expressed in terms of the mean density of positive staining. **(D)** Apoptotic cells were detected by TUNEL staining in tissue sections. **(E)** Semi-quantitative data from the analysis of apoptotic cells. The data are presented as the means ± SD. ^∗∗∗^*P* < 0.001, ^∗∗^*P* < 0.01, ^∗^*P* < 0.05. Each group contained five animals.

**FIGURE 7:**
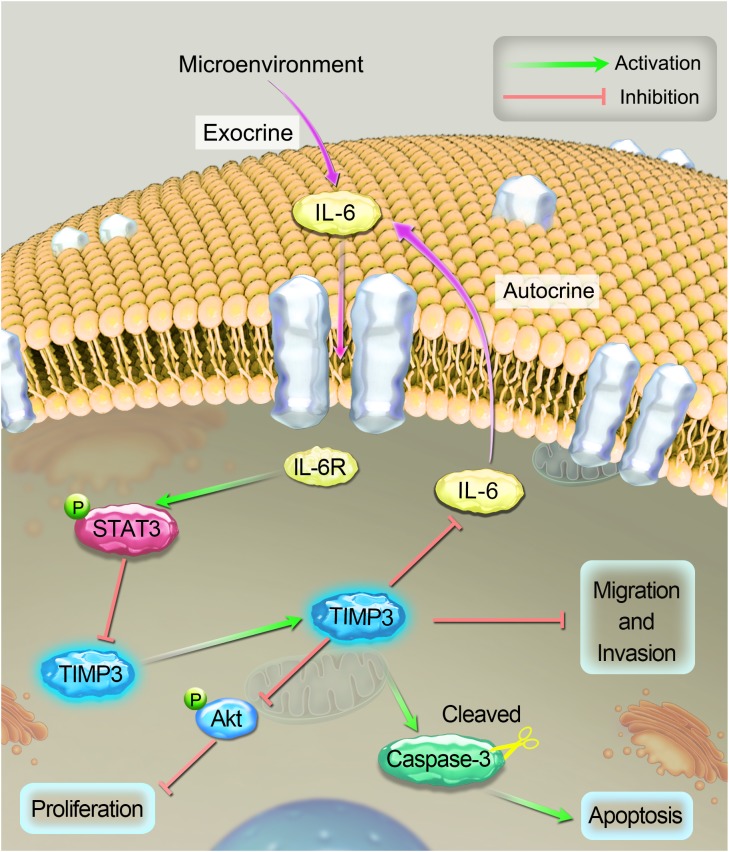
A schematic diagram of the proposed mechanism of cisplatin sensitivity enhancement by TIMP3 overexpression. According to the results of the current study, IL-6 inhibited TIMP3 expression through STAT3 activation, whereas conversely; TIMP3 suppressed the production of IL-6. TIMP3 also facilitates the expression of cleaved caspase-3, while inhibiting the production of p-Akt, which results in the inhibition of proliferation and the overall potentiation of apoptosis. Taken together, these events contribute to enhancing the sensitivity of osteosarcoma to cisplatin.

Tissue sections were prepared from tumors during the 5th week after cisplatin treatment, and the expression profiles of TIMP3, cleaved caspase-3, p-Akt, PCNA, and IL-6 were evaluated using immunohistochemistry. Compared with saline treatment, cisplatin treatment resulted in the downregulation of p-Akt, PCNA, and IL-6, and the upregulation of TIMP3 and cleaved caspase-3 in Saos2-lung tumor cells. In contrast, compared to cells without TIMP3 overexpression, TIMP3 overexpression resulted in substantially increased downregulation of p-Akt, PCNA, and IL-6, and upregulation of TIMP3 and cleaved caspase-3 in tumors of Saos2-lung cells (**Figures 6A–C**). Apoptotic cells were detected by TUNEL staining. We observed that TIMP3 overexpression increased the rate of apoptosis in the osteosarcoma cells treated with cisplatin (**Figures 6D,E**). All these results demonstrated that cisplatin sensitivity correlated positively with TIMP3 expression, which is regulated by the IL-6/TIMP3/Caspase family axis (**Figure 7**).

## Discussion

In this study, we found that IL-6 could inhibit TIMP3 expression in Saos2 and Saos2-lung cells via STAT3 activation. ELISA tests showed that Saos2-lung cells secreted more IL-6 than Saos2 cells. The autocrine effect of IL-6 and the phosphorylation of STAT3 correlated negatively with TIMP3 expression. This was confirmed by STAT3 knockdown. The immunohistochemical analysis of the tumor tissues also showed a negative correlation between IL-6 and TIMP3 expression. In short, IL-6 could inhibit TIMP3 expression through STAT3 activation, while conversely; TIMP3 could suppress the production of IL-6 (**Figure 7**), which is consistent with previous reports, as follows. One study demonstrated that the loss of TIMP3 expression might increase IL-6 production via the tumor necrosis factor α/nuclear factor κB pathway in patients with HPV-infected non-small cell lung cancer (Wu et al., 2012). Another study showed that the serum level of IL-6 was elevated in TIMP3^-/-^ mice (Smookler et al., 2006).

In the present study, we showed that the expression of TIMP3 was positively correlated with cisplatin sensitivity in patients with osteosarcoma. We also investigated the effects of the overexpression and knockdown of TIMP3 on proliferation, chemoresistance, migration, and invasion of osteosarcoma cell lines *in vitro*. The proliferation of osteosarcoma cells was inhibited by TIMP3 overexpression, whereas TIMP3 knockdown had an antagonistic effect. Akt signaling has been reported to play essential roles in the progression of many tumors, including osteosarcoma (Keremu et al., 2017; Liu et al., 2017; Ma et al., 2017), and the PI3K/Akt pathway participates in almost all malignant processes, including tumorigenesis, cancer cell proliferation, metastasis, angiogenesis, and chemoresistance (Yuan and Cantley, 2008; Berlanga et al., 2016; Dai et al., 2016; Davaadelger et al., 2016). Our results showed that the p-Akt level correlated negatively with TIMP-3 expression. This suggested that Akt is a target of TIMP3 in osteosarcoma.

Our results implied that TIMP3 overexpression enhanced cisplatin-induced apoptosis in osteosarcoma cells. This was consistent with the results of our previous study (Han et al., 2016). We further revealed that changes in TIMP3 activity had an effect on the expression and/or activity of caspase family proteins. As shown in **Figure 4**, inhibition of TIMP3 substantially decreased the levels of caspase family proteins, especially cleaved caspase-3 and cleaved caspase-9, after cisplatin treatment. Therefore, osteosarcoma cells showed decreased apoptosis and insensitivity to cisplatin. By contrast, we speculated that the activation of TIMP3 would have the opposite effect on the expression and/or activity of the caspase family proteins, which would increase the sensitivity of osteosarcomas to cisplatin. The results of the *in vivo* cisplatin-sensitivity study and immunohistochemical analysis of the tumor tissues supported this hypothesis. Overall, our study revealed that the expression of TIMP3 correlated positively with cisplatin sensitivity in osteosarcoma. Thus, TIMP3 could be an effective molecular marker for predicting and even regulating the sensitivity to cisplatin in patients with osteosarcoma.

TIMP3 has tumor-suppressive functions in several human malignancies. These effects are mediated via both MMP-dependent and -independent mechanisms, and include the inhibition of tumor growth, angiogenesis, and invasion (Cao et al., 2016; Chen et al., 2016; Das et al., 2016b). A study showed that TIMP3 functions as a tumor suppressor in melanoma, and negatively regulates several aspects of the melanoma metastatic cascade (Das et al., 2016a). Another study showed that lysine (K)-specific demethylase 1A (KDM1A) promoted tumor cell invasion by silencing TIMP3 expression in non-small cell lung cancer cells (Kong et al., 2016). IL-6 is a pleiotropic cytokine that plays a crucial role in cell growth and differentiation; however, its exact role varies and remains unclear (Bian et al., 2010; Wang et al., 2016). Some reports showed that IL-6 is a growth factor involved in breast cancer (Noori et al., 2017), lung cancer (Wang et al., 2017), hepatocellular carcinoma (Lin et al., 2017), gastric cancer (Wu et al., 2017), and ovarian cancer (Ferraresi et al., 2017). Our study confirmed the involvement of the IL-6/TIMP3/Caspase pathway *in vitro* and *in vivo*, and revealed the important role of TIMP3 in cisplatin sensitivity in osteosarcoma. This has implications for the future treatment of osteosarcoma.

However, additional in-depth studies with the following objectives are necessary: First, a large number of patient samples should be collected to further verify the relationship between the IL-6/TIMP3/Caspase pathway and chemotherapy sensitivity. Second, the intermediate molecular mechanisms underlying the link between IL-6 and TIMP3 and the downstream signaling of TIMP3 in osteosarcoma should be better clarified. Furthermore, the relationship between TIMP3 and metastasis should be explored further, and the underlying molecular mechanism should be better described. Finally, other chemotherapeutic drugs with potential capability to induce apoptosis should be tested to reveal the universal mechanisms for such processes.

## Conclusion

Our findings show that TIMP3 levels correlated positively with cisplatin sensitivity in osteosarcoma, which is regulated by the IL-6/TIMP3/Caspase pathway *in vitro* and *in vivo*. Our study provides a deeper understanding of chemoresistance to cisplatin in osteosarcoma, and suggests that TIMP3 could potentially aid in the design of new anticancer drugs and the development of new therapeutic methods to treat osteosarcoma.

## Author Contributions

T-tT, S-hZ, and X-gH: conception and design. X-gH and H-mM: development of methodology. X-gH, H-mM, X-qL, YL, LD, HQ, and Q-mF: acquisition of data. X-gH, H-mM, X-qL, YL, LD, HQ, Q-mF, JZ, S-hZ, and T-tT: analysis and interpretation of data. X-gH and T-tT: writing, review, and/or revision of the manuscript. X-gH, H-mM, X-qL, and T-tT: administrative, technical, or material support. T-tT and Q-mF: study supervision.

## Conflict of Interest Statement

The authors declare that the research was conducted in the absence of any commercial or financial relationships that could be construed as a potential conflict of interest.

## Abbreviations

AMPK: AMP-activated protein kinase
IL: interleukin
MMP: matrix metalloproteinase
STAT: Signal Transducer and Activator of Transcription
TIMP: tissue inhibitors of metalloproteinase.
