# Supplementary material for: TIMP3 Overexpression Improves the Sensitivity of Osteosarcoma to Cisplatin by Reducing IL-6 Production
Source: Front Genet. 2018 Apr 20;9:135. doi: 10.3389/fgene.2018.00135 (PMC5920027; doi:10.3389/fgene.2018.00135)
Supplement: Supplementary file 1 [file Data_Sheet_1.docx]

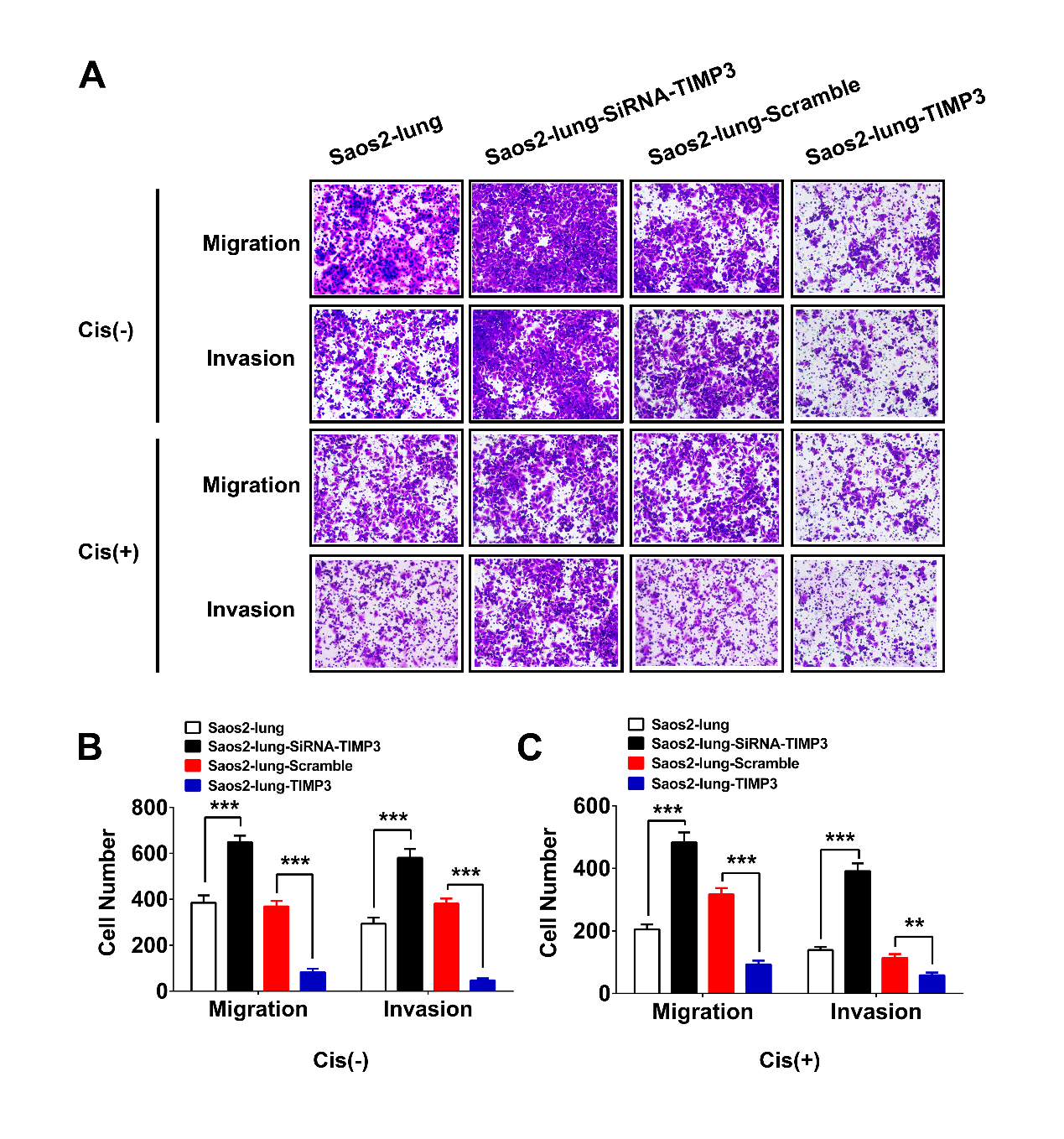


**Supplementary Figure 1.** **Migration and invasion of Saos2-lung cells with or without cisplatin after the overexpression or knockdown of TIMP3.** (**A).** Transfected Saos2-lung cells were cultured with or without cisplatin for 24 h. Then, cells were plated in the upper chamber of a transwell plate, and allowed to migrate for 12 h or invade for 24 h. Cells that migrated or invaded to the lower surface were fixed, stained, and counted. A total of 3–5 random microscopic fields were used for counting cells subjected to each treatment. (**B-C).** The number of migrating and invading cells is the average count of cells in 3-5 random microscopic fields. Data are presented as means ± SD. ***P* < 0.01, ****P* < 0.001. All data were obtained from at least three independent experiments.


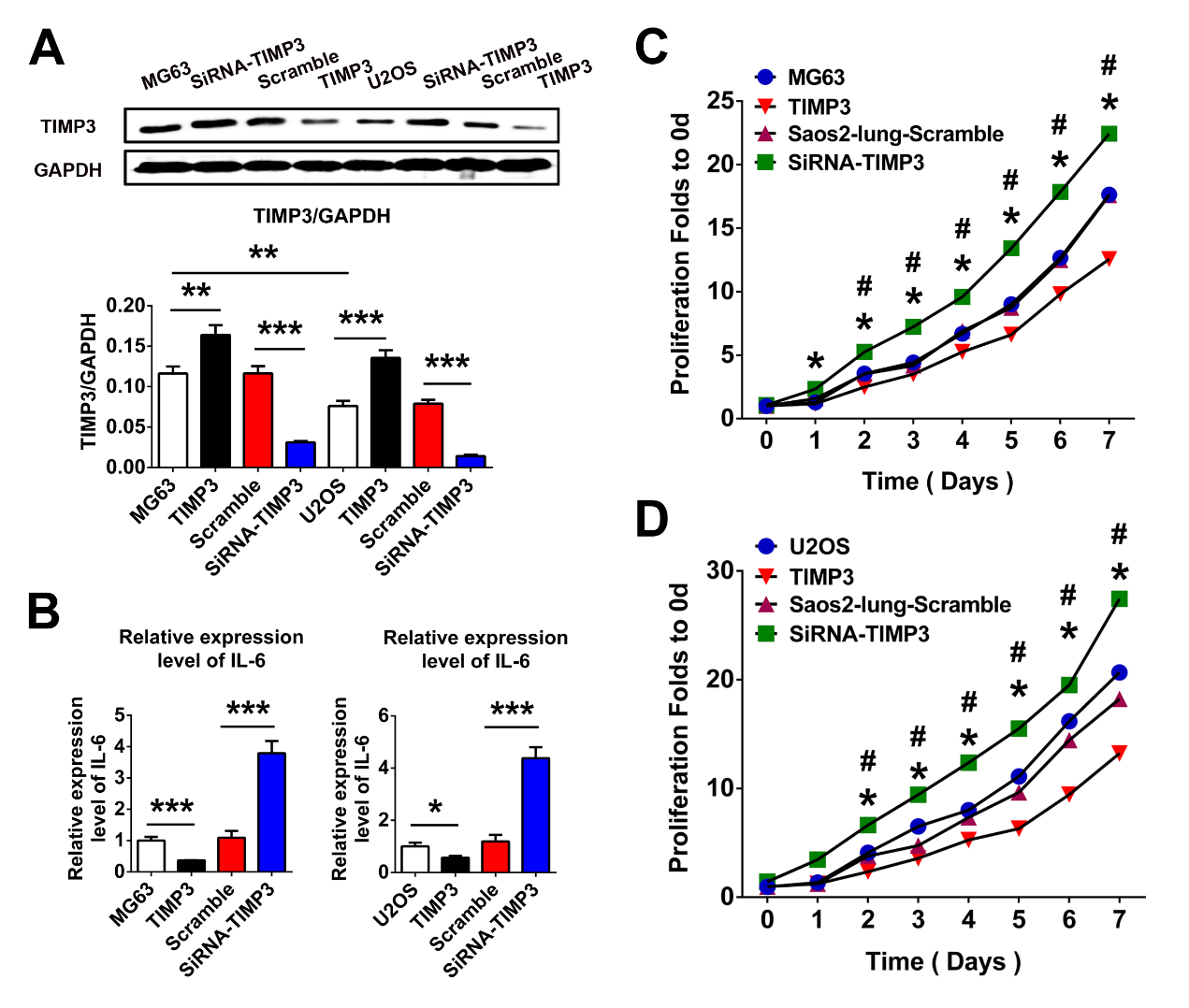


**Supplementary Figure 2. The expression of IL-6 and proliferation in MG63 and U2OS cells after the overexpression or knockdown of TIMP3.** **(A).** The identification of TIMP3 overexpression and knockdown in MG63 and U2OS cells. **(B).** IL-6 gene expression in transfected-OS cells. **(C-D).** Cell proliferation in transfected-OS cells. **P* < 0.05 between MG63 or U2OS and siRNA groups, #*P* < 0.05 between scramble and TIMP3 groups separately in cell proliferation assay. ****P* < 0.001, **P* < 0.05. All data were obtained from at least three independent experiments.


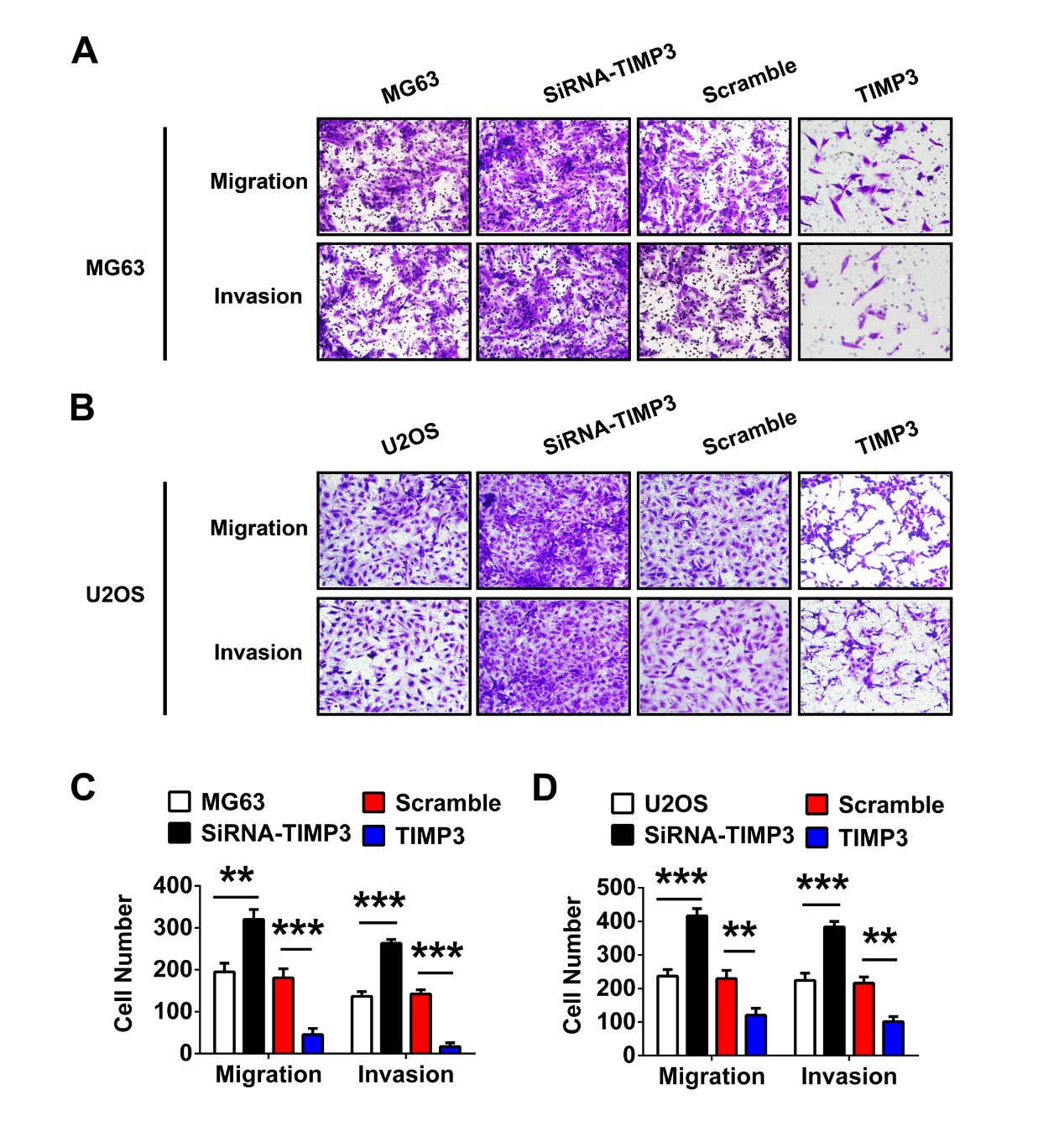


**Supplementary Figure 3.** **Migration and invasion of MG63 and U2OS cells after the overexpression or knockdown of TIMP3.** **(A).** MG63. **(B).** U2OS. **(C, D).** The number of migrating and invading cells is the average count of cells in 3-5 random microscopic fields in MG63 and U2OS cells after the overexpression or knockdown of TIMP3. Data are presented as means ± SD. ***P* < 0.01, ****P* < 0.001. All data were obtained from at least three independent experiments.
